# Long-span fiber composite truss made by coreless filament winding for large-scale satellite structural systems demonstrated on a planetary sunshade concept

**DOI:** 10.1038/s41598-024-58513-w

**Published:** 2024-04-08

**Authors:** Pascal Mindermann, Denis Acker, Robert Wegner, Stefanos Fasoulas, Götz T. Gresser

**Affiliations:** 1 Institute for Textile and Fiber Technologies, University of Stuttgart, Pfaffenwaldring 9, 70569 Stuttgart, Germany; 2 Institute of Space Systems, University of Stuttgart, Pfaffenwaldring 29, 70569 Stuttgart, Germany; 3 German Institutes of Textile and Fiber Research Denkendorf, Körschtalstraße 26, 73770 Denkendorf, Germany

**Keywords:** Coreless filament winding, Fiber composite truss structure, Planetary sunshade, Aerospace engineering, Composites, Characterization and analytical techniques

## Abstract

Climate change necessitates exploring innovative geoengineering solutions to mitigate its effects—one such solution is deploying planetary sunshade satellites at Sun–Earth Lagrange point 1 to regulate solar radiation on Earth directly. However, such long-span space structures present unique technical challenges, particularly structural scalability, on-orbit manufacturing, and in-situ resource utilization. This paper proposes a structural concept for the sunshade’s foil support system and derives from that a component-level modular system for long-span fiber composite lightweight trusses using coreless filament winding. Within a laboratory-scale case study, the component scalability, as well as the manufacturing and material impacts, were experimentally investigated by bending deflection testing. Based on these experimental results, FE models of the proposed structural concept were calibrated to estimate the maximum displacement and mass of the foil support structure, while comparing the influences of foil edge length, orbital load case, and material selection.

## Introduction

Addressing climate change is one crucial engineering challenge for the twenty-first century^1^. In order to mitigate more frequent extreme weather events, intensifying droughts, and $178 trillion by 2070 in estimated total costs caused by global warming, the 2022 United Nations Climate Change Conference^2,3^ has allocated substantial funding for compensatory measures and technological advancements: Whereas carbon capture methods^4^ do not represent a sustainable long-term solution due to their energy requirements and reliance on scarce resources; atmospheric intervention technologies have potential adverse environmental effects.

**Figure 1:**
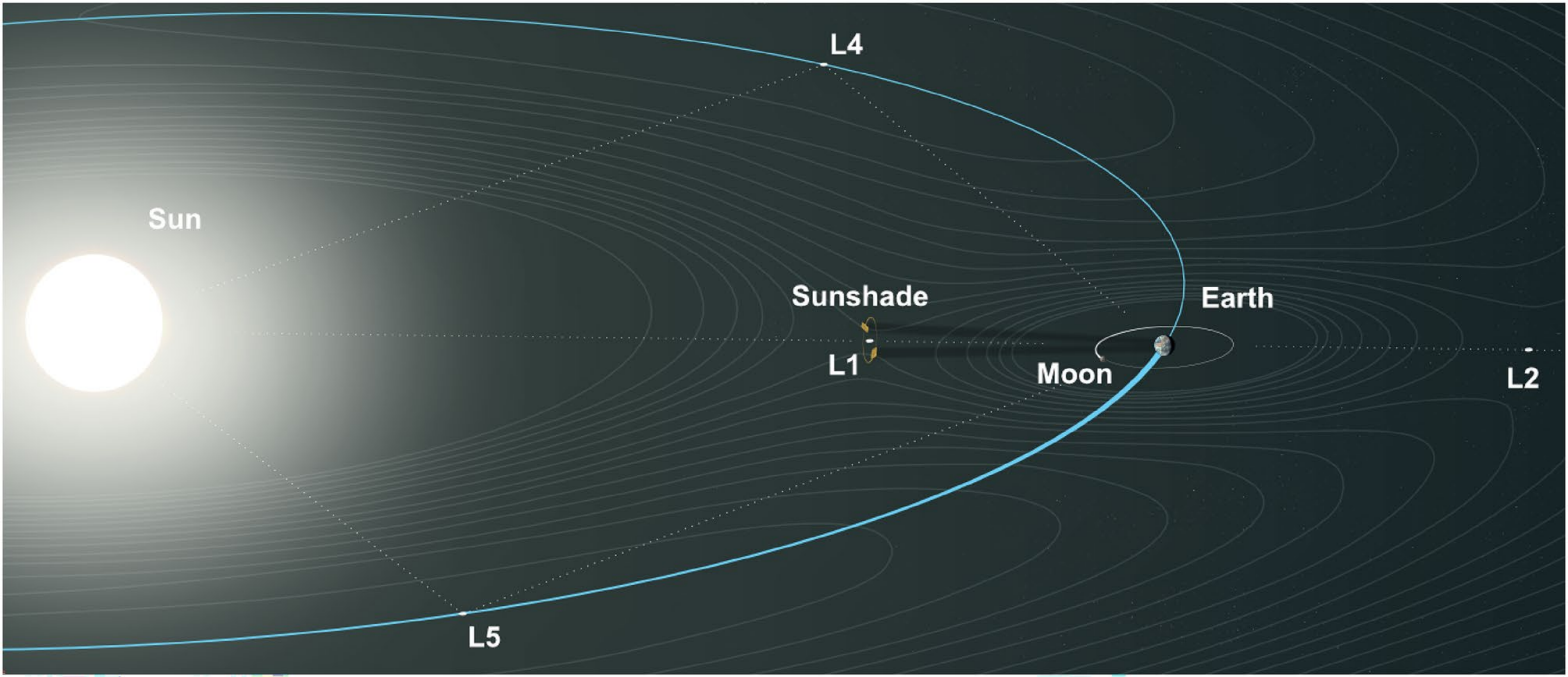
Schematic overview of the mission concept for a planetary sunshade in a halo orbit around SEL1. The sunshade providing shading to Earth. In a later phase of the concept, sunshades are produced mainly from lunar resources. Therefore, manufacturing and maintenance happening at lunar infrastructure, or locally at SEL1.

One promising approach to mitigate global warming involves shading the Earth using a thin foil positioned at the Sun–Earth Lagrange point 1 (SEL1), see Fig. 1. Such a space-based geoengineering method, specifically in the form of the International Planetary Sunshade (IPSS)^5^, is a long-term solution for arbitrarily adjusting the solar constant^6^. Therefore, its direct impact is easy to implement in climate prediction models and does not interfere with ecosystems. To reduce rocket launches the utilization of lunar^7,8^ or asteroid^9^ resources and on-orbit manufacturing^10^ must be conceptualized. Concepts may include three options: prefabricated segmented booms that absorb the tensile forces, rotating satellite system where fiber tension is maintained by centrifugal force, or using lunar surface as a platform. In later stages, a sunshade constellation might potentially provide vast amounts of sustainable energy, and be an initial step towards a Dyson swarm. Energy could be delivered via wireless power transmission^11^ to Earth, the Moon and other destinations. However, the design of such a solar energy satellite is technically much more complex than a mere passive sunshade. Prior research^12^ suggests that to limit the average global surface temperature rise to 1 K, a sunshade area of nearly 1.7 million km^2^ is required. As fabricating at such scales present immense structural as well as logistical challenges, the focus shifted to concepts aggregating or connecting a multitude of individual sunshades with smaller foil sizes, for example on the other end of the spectrum with only 1 km^2^. Yet the optimal size must be defined by the trade-off between complexity in maintaining the orbital constellation and fabrication restrictions. Trusses represent an optimal choice for supporting structures for the foil owing to their inherent lightweight potential, redundancy, scalability, and adaptability. In addition to conventional metal trusses, assembled from individual components, integrally fabricated fiber composite trusses exhibit enhanced lightweight capabilities attributed to decreased connecting elements. Overcoming the current fabrication constraints for such large-scale lightweight aerospace structures, specific to the sunshade or in more general, necessitates the utilization of novel fabrication methods, such as coreless filament winding (CFW).

CFW is an emerging additive manufacturing technique with the potential for fabricating high-performing and efficient fiber-reinforced composite structures^13–15^. In comparison to other manufacturing processes such as conventional filament winding, pultrusion, or continuous fiber-reinforced 3D printing, CFW offers distinct advantages including adaptability at low investment cost, geometrical design freedom, and high processing speeds^16^. In common practice, building elements often demonstrate topologies of either shells^17^ or lattices^18^. CFW eliminates the need for a mandrel or mold of conventional filament winding, as fibers are spanned freely between anchors mounted to a winding frame. The composite material is not supported over large areas but only has point-like contact with the anchors. A continuous fiber bundle impregnated with a synthetic resin is spanned freely between these anchors in a specific sequence, the winding syntax^19^. As an ordered list of anchors, the winding syntax represents both a manufacturing instruction and a geometric description of the structure. In addition, the hooking syntax defines how the fiber is hooked at each anchor. Fibers are usually impregnated online via a resin bath or cartridge-based system^20^. The most common anchoring approach involves a winding pin^21^, consisting of a metallic sleeve with washers at both ends bolted onto a winding frame. The fiber composite only contacts the sleeves, which typically remain within the structure after removal from the fixture. In some cases, hybrid forms of CFW incorporate auxiliary surfaces to support fibers. Most often, the fiber tension is controlled by a passive mechanical system during winding. The ability to individually place each fiber bundle provides complete control over the syntax, enabling the customization of the fiber net, resulting in high mechanical performance at minimal material usage and allows for the creation of free-form components without significant equipment modifications. Moreover, the CFW process can be fully automated using industrial robots^22^, potentially enabling on-orbit manufacturing. However, also traditional filament winding machines can be used for CFW when implementing a winding fixture. Winding frames^21^ offer rapid reconfiguration without requiring high surface accuracy or release agents, distinguishing them from mandrels or molds. However, due to the absence of guide surfaces, CFW lacks consolidation^23^ and experiences variations in fiber tension^20^. These effects significantly impact the fiber-fiber interaction, influencing the mesoscopic fiber net configuration and the inner and outer fiber bundle structure^24^. As a result, accurately predicting and controlling the structural properties of the final component becomes difficult. Designing with CFW therefore requires meticulous effort involving finite element simulations^25–27^, as even minor changes to the fiber net can have a substantial impact on its structural performance^28^. Nevertheless, CFW has demonstrated its potential in producing adaptive load-optimized structures across different scales, benefiting areas such as architecture^29,30^, automotive^31,32^, and aerospace^28,33^.

Previous research investigations^34–36^ and commercialization efforts^37–39^ have primarily focused on conventionally filament-wound fiber-composite trusses for lightweight applications. However, these structures often required extensive framing, sometimes spanning the entire component length, and additional elements such as wrapping tape, metallic supports, pultruded rods, or prefabricated rings. On the other hand, the scaling-up of entirely free-spanning CFW trusses has been explored^40^, revealing that maintaining positional control over the fiber during the winding process leads to a disproportionate increase in fiber tension. Another area of current research in CFW involves expanding the range of material systems. In addition to carbon and glass fibers with epoxy resin, successful implementation of mineral fibers has been achieved^41^, offering the potential for sourcing materials from in-situ resources^42^. Furthermore, several case studies have investigated the impact of fabrication deviation^43,44^ and correlated data across different process domains^45^. Given that load capacity of CFW structures is significantly influenced by point load induction at anchor elements, various anchor configurations have been studied^46–48^. The transfer of point loads into fiber composites poses a challenge, mainly due to elevated stress concentrations. In response, methods for measuring the distribution of such loads have been developed^49^. Despite these advancements, winding pins^21^ involving a smooth metallic sleeve between two washers on a bolt still present several disadvantages for the current application.

This paper aims to propose a structural system concept for a global sunshade design. From that, load cases on component level will be derived, leading to a truss design. This will allow several experimental investigations: Initially, the feasibility of the CFW manufacturing method will be proven for the different-sized trusses in laboratory scale. Then, to explore the scalability of the trusses, components with 5, 10, and 15 m form-factor will be investigated. Fabrication deviation on component level and the impact of the material system will be quantified on the 5 m form factor. The experimental results will be used to calibrate numerical simulations, which will then allow accessing the structural performance of large-scale trusses made from lunar material and, thus, finally evaluating the proposed CFW structural concept for future application in sunshades and other large-scale satellite systems.

## Conceptual design of the sunshade structural system

Even though the emphasis of this study is on the investigation of the truss system, the sunshade’s global structural system should not remain unmentioned. Here, a potentially viable concept envisions an evolutionary, modular, and scalable sunshade design with incremental development stages spanning several decades: In the initial phase, sunshades are launched from Earth utilizing unfolding mechanisms^50–53^. Subsequently, in-space manufacturing and assembly (ISMA) technologies^54^ must be applied, considering modularity and segmentation of foil support truss structures^48^. In later stages, the sunshade system’s material is increasingly replaced by in-situ resources (lunar materials).

### Load cases

The load cases for several potential sunshade’s orbits were analyzed, focusing on the atmospheric drag F_D_ and solar radiation pressure F_sp_, see Table 1. Flights in low Earth sun-synchronous orbits (SSO), in Earth geosynchronous orbits (GSO), and SEL1 halo orbits are considered. For later calculations, it is assumed that the atmosphere and the solar radiation hit the satellite from the front and normal to the foil surface. While this is realistic for the solar pressure, it represents an extreme case to be avoided for the atmospheric drag. In both cases, an even load distribution of the foil is anticipated.

**Table 1:** Load cases, including atmospheric drag F_D_ and solar radiation pressure F_sp_.

Orbit type	Orbital altitude [km]	Sunshade edge length [m]	F_D_ [N]	F_sp_ [N]
SSO	1000	200	8.31	0.331
		500	52.00	2.082
		1000	208.00	8.301
GSO	35,850	200	0.00	0.332
		500	0.00	2.083
		1000	0.00	8.303
SEL1 halo	1,500,000	200	0.00	0.347
		500	0.00	2.114
		1000	0.00	8.476

### Form finding

**Figure 2:**
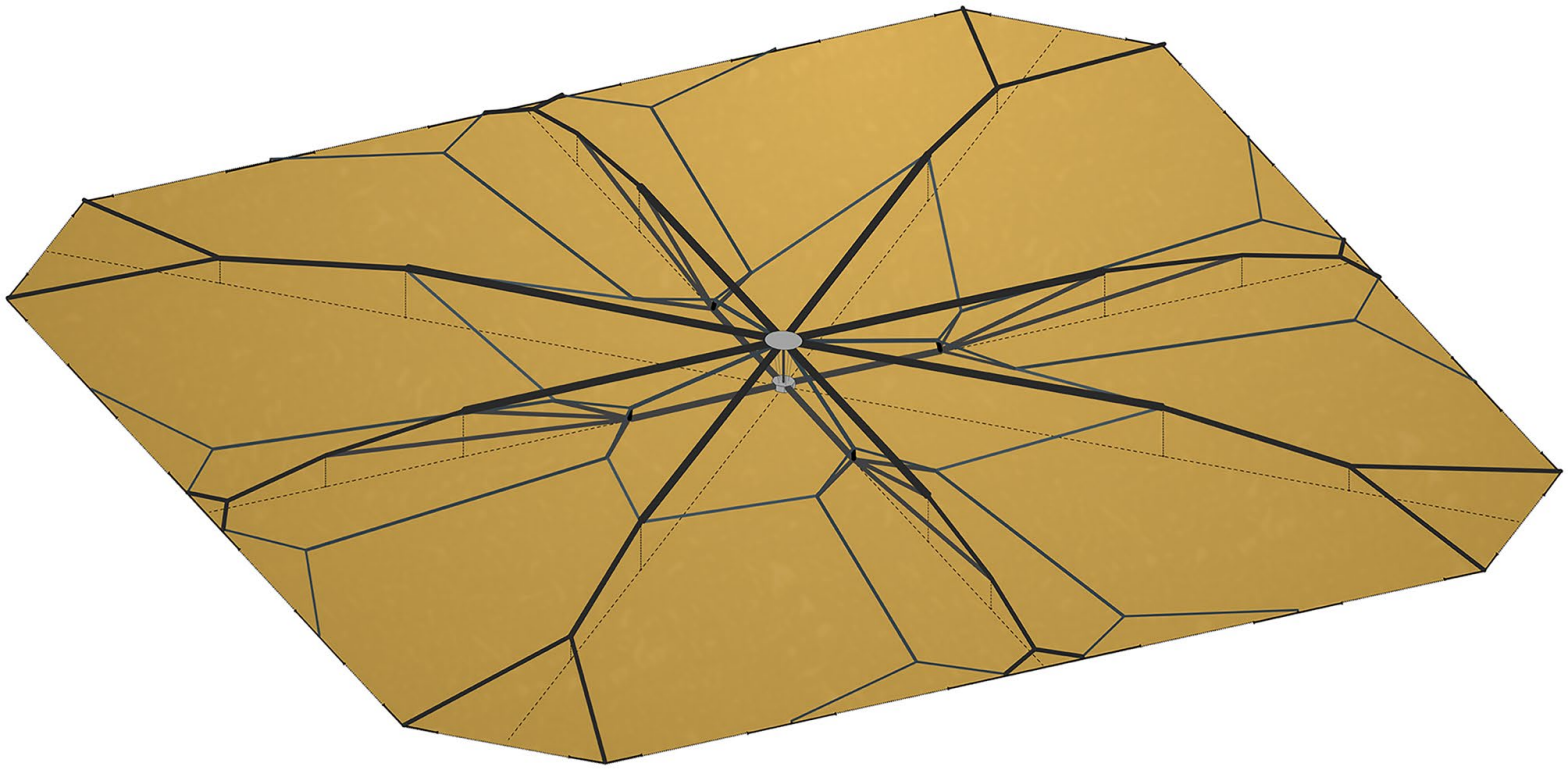
Orthographic rear view of the planetary sunshade satellite’s structural concept with a height to edge length ratio of 4/100. The front side of the satellite is pointing downwards (towards the sun). Solar radiation pressure is applied in the opposite direction. The central hub in light gray, trusses in different darker gray tones, and dashed guide lines shown only for better optical differentiation.

The design methodology employed for the truss structure of the planetary sunshade satellite system encompasses a multi-step procedure, including numerical simulations. Initially, a foundational configuration is established, wherein the majority of subsystems are located within a central hub situated at the rear of the satellite, while a single shading foil with a perimeter in the form of a polygon is intended to be suspended flat on the satellite’s front side. To accommodate this arrangement, a support structure is devised, located exclusively behind the foil and limited to 4% of the foil’s edge length in the dimension perpendicular to the foil. The design process starts with defining the cuboidal build space, allocating the load at the foil’s edge, and setting the nodes located at the central hub as a fixed support. Subsequently, a soft kill option (SKO) topology optimization^55^ generates a volume-based model that represents the force flow. For simplicity, isotropic material properties were assumed during the early stages of the design process. In the final step, this model is interpreted as a symmetric configuration of straight trusses, see Fig. 2. The generated undirected graph (nodes and segments) is assembled from different raw SKO output features. To increase efficiency and redundancy, these trusses may themselves have a sandwich configuration and may also be subdivided along their main axis^48^. In any case, the individual truss segments’ load-carrying capacity must reflect the varying cross-sectional characteristics observed in the raw SKO output.

## Case study in laboratory scale

Based on the segmented design of the structural sunshade foil support system, an experimental investigation was carried out in laboratory scale to explore the scalability of components, as well as the impacts of manufacturing and material properties. Due to the inherent challenge of mitigating the effects of gravity during both production and testing phases, numerical simulations must be employed to deduce insightful findings regarding full-scale trusses in the frame of the before-mentioned load cases of sunshade applications.

### Conceptual design of the truss system

The truss’s component-level design aims to achieve a balance between high mass-specific structural stiffness and low manufacturing complexity. Since autonomous manufacturing in space is envisioned, the avoidance of manufacturing aids and metallic inserts is prioritized, and the material usage is targeted towards the lower end of the weight per stiffness spectrum. Therefore, the simplest geometric primitive, an equilateral triangle, is chosen as the base shape for the truss^48^. A high mass-specific stiffness requires high-performance materials, and in case of bending, a framework configuration is favorable as it allows for positioning material cross-sections further away from the neutral axis.

**Figure 3:**
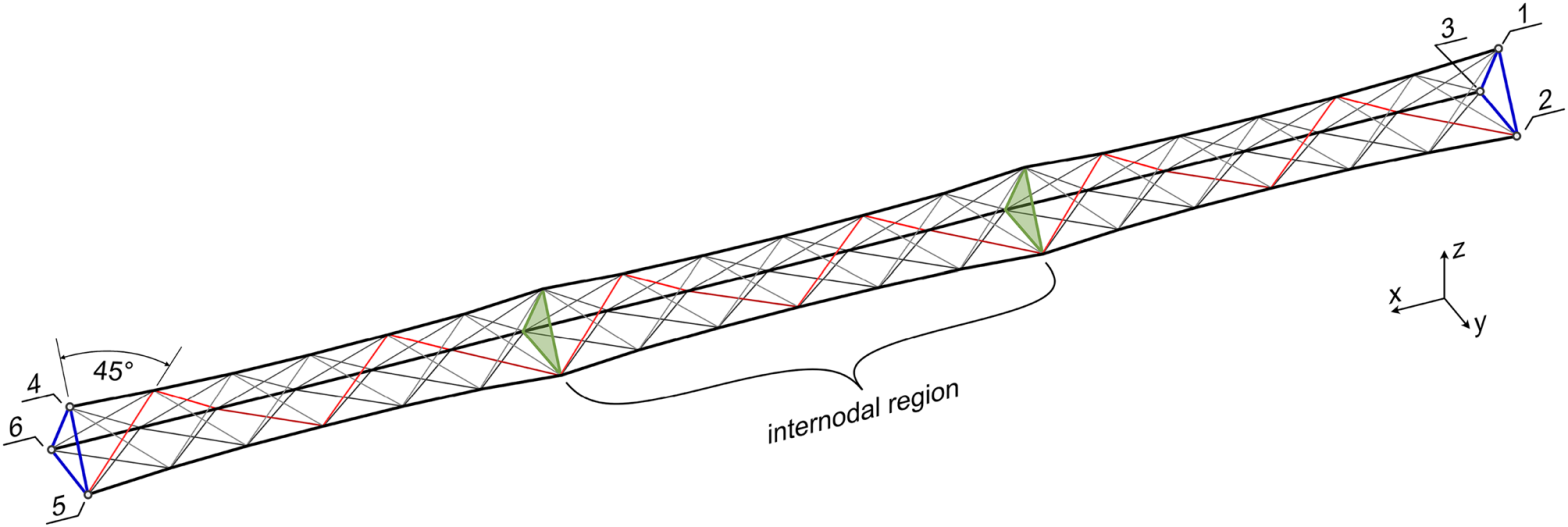
Structural concept of a truss demonstrated for the 5 m form-factor, consisting of three internodal regions. Designation of the nodes (1 to 6) is identical for all form-factors. Regions of the fiber net: main fiber strands (black), lateral cross-bracing (gray), nodals (green), and edge reinforcement (blue). One sequence of the wrapping subsyntaxes is highlighted in red.

Three fiber strands located at the corners of the equilateral triangle form along the main axis of the truss its primary top and bottom chords. These main strands are wound first, and then wrapped multiple times from the outside without interweaving. This wrapping process creates a lateral cross-bracing, which introduces shear members and forms the main strands into a coherent structural unit. Torsional loads are absorbed by utilizing 45-degree wrappings originating from each of the three winding anchors. This results in six fiber strands forming the complete lateral cross-bracing. Unlike existing methods, the rovings used in this design are not subjected to twisting during winding^35^. Due to the fiber–fiber interaction and fiber tension the wrapping introduces a necking of the three main fiber strands. Reducing the number of wrappings reduced necking but leads to asymmetry, which in turn induces adverse torsion under bending conditions. Although a multi-stage winding process^43^ is conceivable for later applications, it was not employed for the case study, due to reduced interfacial bondings and increased production time.

**Figure 4:**
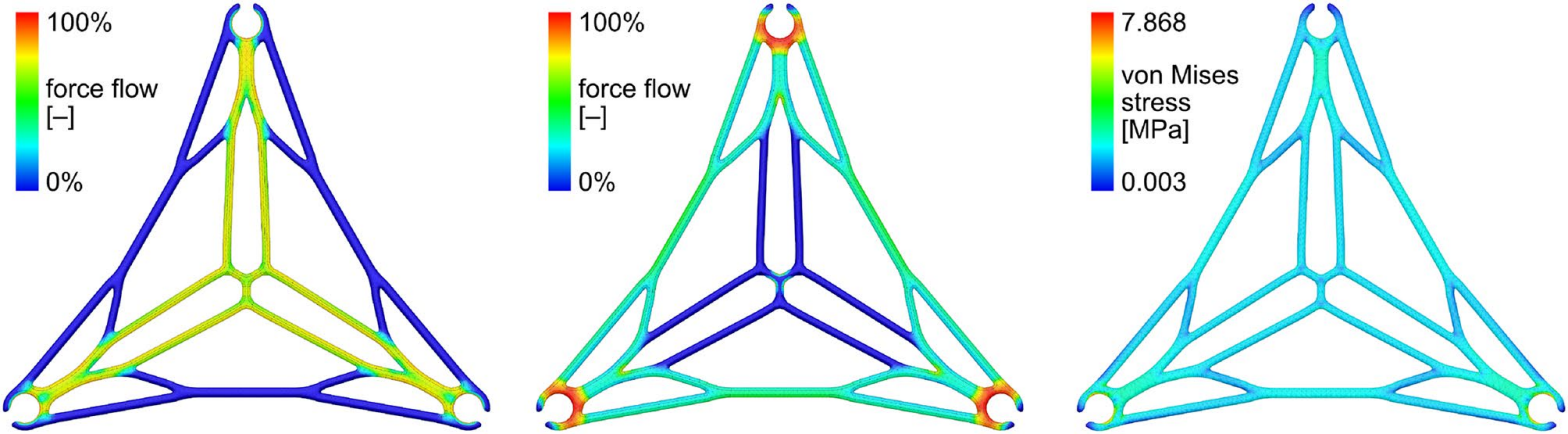
Finite element analysis of the nodals. Left: Force flow analysis for a localized load induction. Center: Force flow analysis for a planar load induction. Right: Stress distribution for load induction of 200 N total.

To address the issue of necking, spacers in the form of internally placed nodal diaphragms, referred to as nodals, are inserted with a side length of 30 cm. In the case study, additively manufactured nodals made of acrylonitrile butadiene styrene (ABS) were deployed, for later application, CFW may be used to prefabricate these nodals. To minimize the mass of the nodals while ensuring they can withstand the compression forces, an SKO topology optimization was applied. After finalizing the design, the geometry was assigned a 1 mm wall thickness, halving the overall mass. Additionally, a computer aided optimization (CAO) was performed mainly improving the fillets of the structure. The final structure was then subjected to static finite element simulation for evaluating its structural performance, as shown in Fig. 4. The stress, except peak at load induction, remains well below 3 MPa. Nevertheless, such a high safety factor is necessary as the load induction is not perfect.

Considering the relatively high fiber material stiffness, even of the polyacrylonitrile (PAN) carbon fibers, it is necessary to incorporate edge reinforcements as a first and last layer and at both ends of the truss to distribute a potentially uneven load induction^49^. In contrast to state-of-the-art sin–cos edge reinforcements, separated inner and outer edge reinforcements, with full wrapping, were selected, avoiding elevated deflection angles at the washers of the winding pins. The case study employs winding pins in the form of a sleeve-washer combination with threaded sleeves. However, future applications may utilize clamps^48^ as winding anchors, positioned on a tilted plane relative to the truss’s main axis. This configuration would enable angled connections between trusses.

The resulting structural system allows for stepwise scaling, while continuous scaling would alter the desired 45-degree wrapping angle. Together with the winding pin and material parameters, the manufacturing instructions of this structural system are fully described by the truss’s base side length, the distance between nodals, the material distribution in the main strands, lateral bracings, and edge reinforcements. Although space and case study trusses may deviate in these parameters, no conceptual changes are anticipated, making the case study truss in term of the scope of investigations planned a suitable proxy for the space truss system.

### Material system

The material system utilized for the samples of the case study includes PAN- and pitch-based carbon fiber rovings, a pitch-based carbon fiber reinforcement tape, a three-component epoxy system, and metallic sleeves. For the production, Teijin Tenax-E HTS40 E13 24K^56^ with 1600 tex at 1.77 g/cm^3^ with a nominal tensile modulus of 240 GPa was used as PAN-based carbon fiber rovings and Mitsubishi K13916^57^ with 16K and 2200 tex at 2.15 g/cm^3^ with a nominal tensile modulus of 760 GPa was used as pitch-based carbon fibers, both as roving and tape. The tape measures 15 × 0.1 mm and includes a polyester binder on one side. The epoxy system MGS LR635, MGS LH635, and MGS LH637 by Westlake^58^ was selected as matrix in a 100:10:20 ratio to achieve high stiffness while avoiding temperature treatments. The definition of a space-suitable material system requires further research especially in terms of ISMA, where UV-triggered curing could be a promising approach for thermoset resins. The sleeves of the winding pins remain within the component and are M12 round zinc-plated steel connecting nuts.

The material distribution was set with the help of a simple mechanical model representing the truss cross-section. The model was input the given geometrical conditions of the winding setup and the expected loading during handling and testing. For example, the 10 m form-factor truss was expected to have a self-weight-induced tip deflection of 120 mm when clamped only on one end. With a 10 N loading at the tip, an additional deflection of about 100 mm was expected. It should be mentioned that increasing the material usage does not improve the theoretical stiffness of the truss when loaded by its dead weight, only if external loads are applied. Therefore, a high fiber volume ratio (FVR) is favorable for such structures.

### Sample fabrication

**Figure 5:**
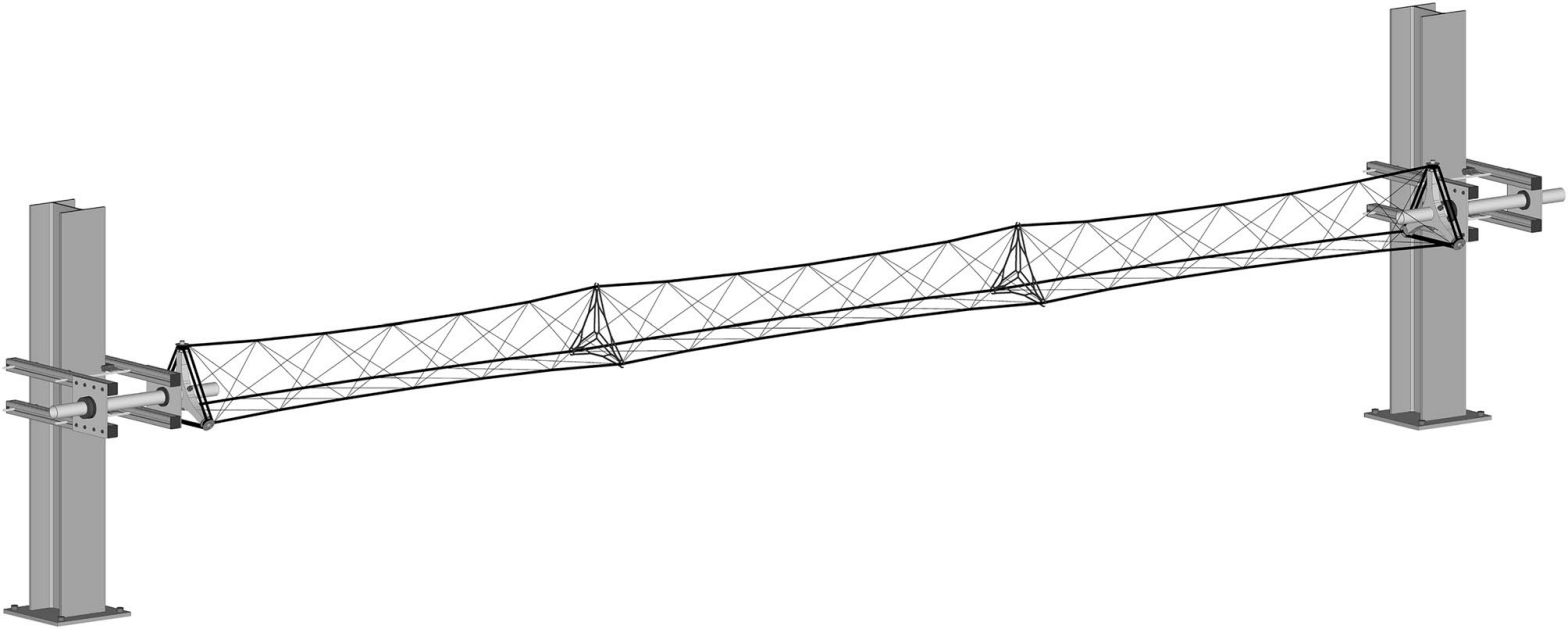
Winding setup. Winding frames clamped via support structures to the H-beams of the laboratory building in a 5 m form-factor configuration. Truss can be rotated around its main axis.

The three required winding pins per cluster were radially bolted in place to a triangular carrier milled from aluminum. To minimize any relative motion between the fiber composite and the bolt, especially during stiffness measurements, the winding pins utilize threaded sleeves instead of state-of-the-art smooth ones, see Fig. 7. To keep the winding setup, see Fig. 5, requirements minimal and considering the length of the component, the winding frames were attached to two of several H-beams in the laboratory building using support structures assembled from aluminum profiles. H-beams with an irregular grid spacing of approx. 5 m were available. ABS bearings were used to mount the frames to the supports, allowing free rotation and facilitating fiber deposition, especially during the cross-bracing winding syntaxes. Since both frames on each side of the setup were not coupled, the synchronous rotation was performed manually by two operators. Due to the given spacing of the H-beams, the actual component lengths differ from the nominal form-factor length values. Additionally, a table trolley was available to carry a spool holder, fiber source, and premixed matrix in separated containers, particularly for longer winding sessions. Fiber tension was controlled manually, while the operator used a hand-held device for impregnation and fiber placement. To achieve the highest possible FVR, a second operator was involved in the winding process of the three main strands, performing manual consolidation to remove excess resin and reduce the formation of void. The winding and hooking syntaxes for an entire component of the case study are provided in Table 2, emphasizing the functional separation between, edge reinforcement, main syntax, and wrapping. Nodals were inserted after the first cross-bracing layer was wound. Following the deposition of fibers, visual inspections were conducted, and individual adjustments to fiber tension were made to achieve consistent internodal necking. In cases where pitch tape was applied, the final step involves manual tape placement by two operators. The tape was placed on the other sides of the fiber bundles and wrapped around the pins for better load induction, followed by impregnation using a resin-saturated paintbrush. The samples were removed from the winding frame after mechanical testing, whereby the sleeves remained inside the structure.

**Table 2:** Winding and hooking syntaxes for case study trusses with variable length.

Syntaxes name	Winding syntax	Hooking syntax
Edge reinforcement, left side, inner	*10* × [4, 5, 6], 4	Full wrap (H = 1)
Bridge from the left to right cluster	4, 1	–
Edge reinforcement, right side, inner	*10* × [1, 2, 3], 1	Full wrap (H = 1)
Bridge from the right to left cluster	1, 4	–
First main fiber strand	4, *9* × [1, 4]	Half wrap (H = 0)
Bridge to next main fiber stand	4, 5	–
Second main fiber strand	5, *10* × [2, 5]	Half wrap (H = 0)
Bridge to next main fiber stand	5, 6	–
Third main fiber strand	6, *10* × [3, 6]	Half wrap (H = 0)
Bridge to the start of the wrapping	6, 4	–
Wrapping for the cross-bracing	4, 1*, nodals, 4*, 5, 2*, 5*, 6, 3*	Full crossing wrap (H = − 1)
Edge reinforcement, right side, outer	*10* × [3, 2, 1], 3	Full wrap (H = 1)
Wrapping from the right to left cluster	6*, 4	–
Edge reinforcement, left side, outer	*9* × [4, 5, 6], 4	Full wrap (H = 1)
Optional reinforcement tape placement	4, 6, 3, 1, 4, 5, 2, 1	Full crossing wrap (H = − 1)

The asterisk indicates that the node is not directly approached but via a 45^∘^ wrapping along the truss, see red line in Fig. 3. Numbers in italics indicate repetitions. Doubling of node indices when stacking the subsyntaxes must be ignored. The keyword *nodals* in the wrapping subsyntax indicates the moment for the insertion of the nodals, see Fig. 4. Hooking condition H as defined in Fig. 7 of^21^.

### Testing procedure

**Figure 6:**
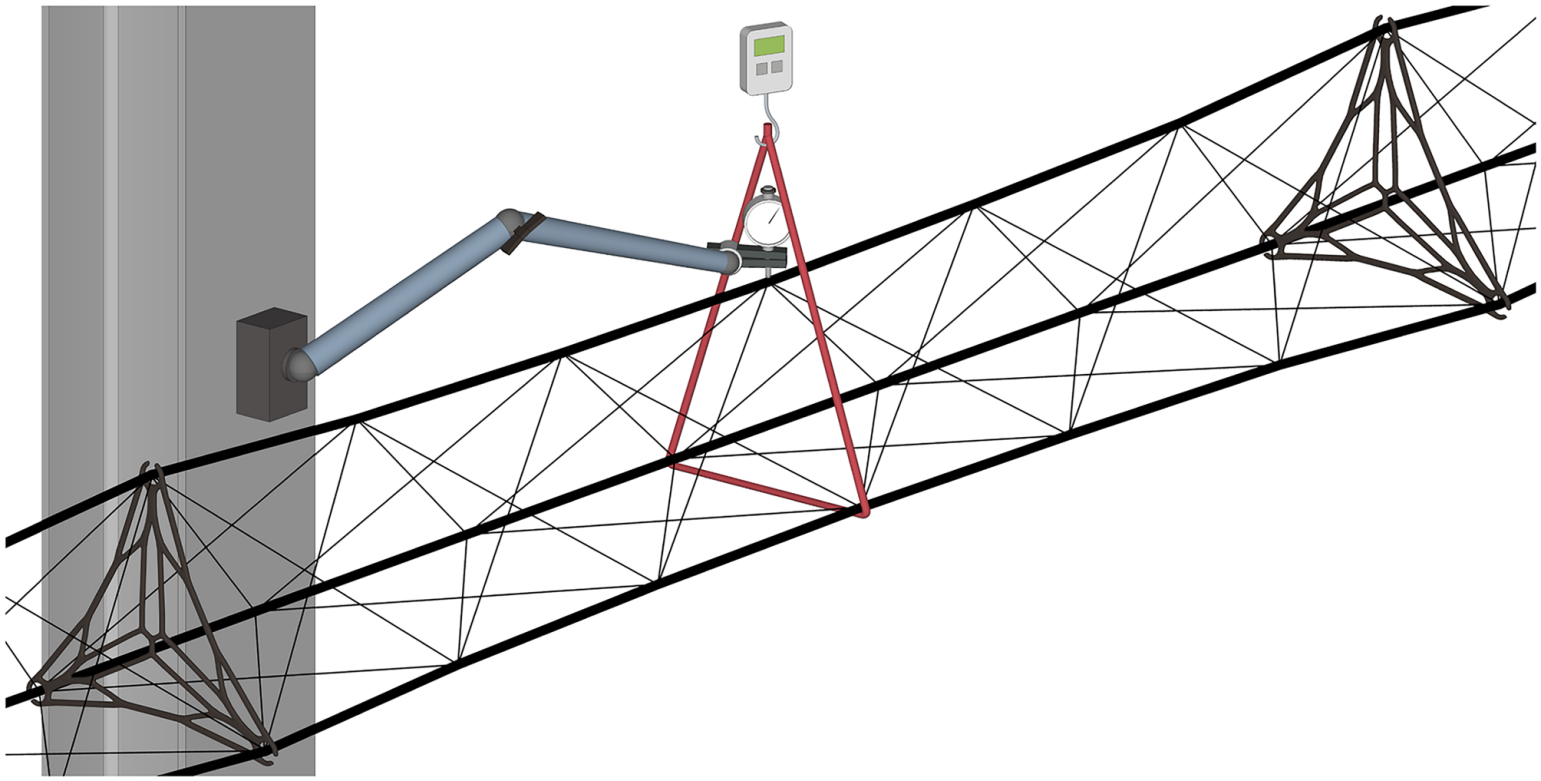
Testing setup of the non-standardized 3-point bending test. Truss was fixed on both sides at all threaded sleeves, see Fig. 5. The red string represents a dry carbon fiber loop connected to a digital spring scale, which was pulled upwards. The dial gauge was attached via an adjustable lever to an external fix point, e.g. an H-beam.

Considering that the component cannot undergo axial tension or compression testing in the laboratory until it reaches the point of global failure, the focus was exclusively on conducting bending deflection testing to obtain insights into the structural stiffness, see Fig. 6. A non-standardized 3-point bending test was used, where the component was fixed on each side at all threaded sleeves. The components’ triangular cross-section was facing upwards and was maintained since the beginning of the resin curing phase to avoid any intervening handling. An upwards tensile load was applied without preloading. The load was applied at the midpoint of the component length to both lower beams via a carbon fiber loop to minimize elastic deformation. The deflection was measured by an analog dial gauge at the upper beam opposite both load induction points. Multiple measurements were taken with increasing deflections. In the case of local buckling, the measurement was discontinued at that force level.

## Results and discussion

**Figure 7:**
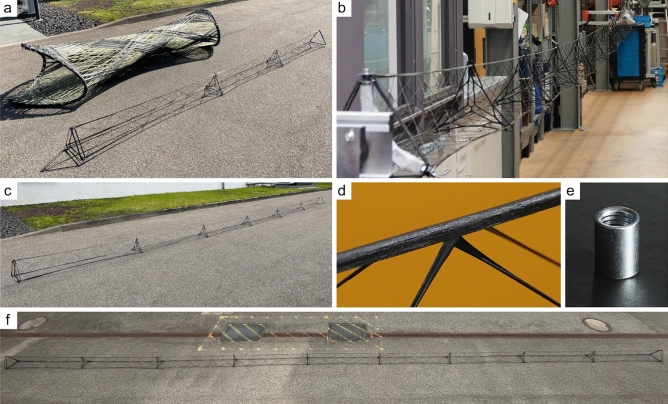
Photos from the case study. (**a**) 5 m form-factor truss in size comparison with a BUGA Fibre Pavilion C5 component^59^. (**b**) 15 m form-factor truss after fabrication. (**c**) 10 m form-factor truss. (**d**) Interaction between the pitch tape and the cross-lateral bracing. (**e**) Threaded sleeve of a winding pin. (**f**) 15 m form-factor truss.

The case study was limited to six samples due to the considerable effort required for the manual sample fabrication, see Table 3 and Fig. 7.

**Table 3:** Overview of the produced and tested sample components of the case study.

ID	Sample description	Form factor (m)	Roving	Tape	Nodals	Objective/Section
1	5 m (no tape)	5	PAN	No	2	Impact of the tape reinforcement
2	5 m (1)	5	PAN	Yes	2	Fabrication deviations
3	5 m (2)	5	PAN	Yes	2	
4	5 m (pitch, no tape)	5	Pitch	No	2	Impact of the pitch rovings
5	10 m	10	PAN	Yes	5	Impact of the scaling
6	15 m	15	PAN	Yes	8	

### Fabrication implications

**Figure 8:**
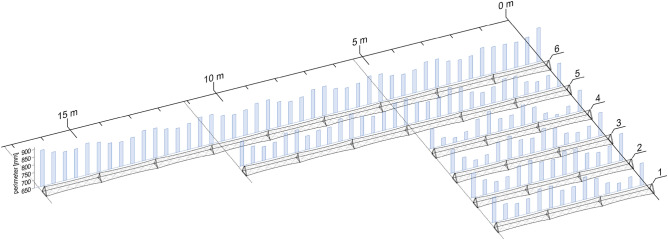
Necking and length comparison for the different trusses with 5.3 m for samples 1–4, 11.1 m for sample 5, and 17.3 m for sample 6. Necking is measured as the truss’s perimeter along the truss and plotted as a bar chart behind each sample model. Smaller values correspond to stronger pronounced necking where 900 mm equals no necking.

The consistent execution of individual work steps by the same operator was a key strategy to minimize unnecessary deviations in the winding process. As the fiber tension substantially influences the CFW process, this parameter was the most crucial aspect addressed by this approach. As the operators and winding frames were unaffected by the fiber tension, even when working on the longer samples, a future on-orbit fabrication seems more feasible than anticipated. For the fiber net configuration of the trusses, the fiber tension directly results in fiber–fiber interaction, generating the necking phenomenon, see Fig. 8. The necking was least pronounced for sample 6 with an 835 mm average truss perimeter, whereas the strongest necking can be found on sample 4 with 12% less. Assuming equal tension during wrapping, this would correspond to the lower tension when using the pitch fibers. The other 5 m form-factor samples’ perimeter is similar and averages 760 mm. Despite being longer, the 10 m sample does not exhibit equally decreased necking with a 774 mm truss perimeter on average. It could also be found that for the 5 m form-factor samples the necking is less pronounced at the central internodal region. The fiber tension can consequently be measured indirectly via the pronouncedness of the necking, but slight differences cannot be resolved. Notably, higher fiber tensions did not hinder impregnation quality, even at increased deposition speeds. However, when transitioning to different material systems, restrictions may arise. Due to gravity, global sagging of the trusses was observed depending on their dead weight and length, see Fig. 7b. In order to counteract sagging, a higher fiber tension was applied to the main fiber strands for longer form factors. This also caused a decrease in necking, which can be compensated by increased fiber tension during the wrapping syntax. Additionally, the introduction of nodals increased the dead weight, which amplified the sagging. Thus, minimizing the nodals’ mass is crucial for future applications. Pre-buckling of fiber segments was prevented by completing each sample within a single winding process and using a sufficiently long pot life of the resin system. Utilizing CFW for nodal prefabrication streamlines logistics and ensures material system consistency. Inserting the nodals, adds friction to the wrapping syntax, which reduces tension-compensating movements. Consequently, the balancing of the necking occurred primarily within the internodal regions, leading to differing degrees of necking between adjacent internodes. Manual intervention compensated necking issues by adjusting the fiber length of the wrapping, emphasizing the significance of tension control in subsequent automated setups.

No adjustments to the equipment or process were made to accommodate the unique characteristics of pitch fiber carbon during winding. Nevertheless, the pitch carbon fiber rovings could be processed as the operators used a novel winding method^23^, employing trajectories that minimize filament damage during all winding phases. However, the primary difference compared to PAN carbon fibers is the significantly higher susceptibility to shear forces, which still requires careful attention to maintain a large nozzle–fiber angle, especially during hooking phases. Therefore, the general fiber tension was lower compared to the PAN-based samples. Future adjustments to the winding equipment could increase the applicable fiber tension, potentially resulting in higher structural stiffness. As the same nozzle diameter was used for both roving types, the deposited pitch fibers were visibly drier due to 14% more dry-roving cross-sectional area. This was evident through the absence of resin dripping during the winding of the pitch rovings and by an increased fiber mass ratio based on material consumption. A higher FVR should result in more structural stiffness until an excessively high FVR compromises roving-to-roving bonding and structural performance. Insufficient bonding occurred only in the edge reinforcement syntax, caused by strongly reduced fiber tension to avoid shear failure at winding pin washer edges. This was irrelevant to the testing as the edge reinforcement was fully clamped. The optimal FVR in CFW depends on the material system and the component geometry considering the anticipated load cases.

Even when subjected to high fiber tension during draping, the pitch reinforcement tape demonstrated excellent processability, despite its considerable fiber fuzz. So, it could also be wrapped around winding pins and was perceptibly less susceptible to shear forces than the pitch fibers. As the fiber bundles of the main strands were still wet during draping, the tape bonding occurred directly. The laterally extending tape conformed to the elliptical cross-section of the roving bundle, facilitating easy folding of the tape into a V-shaped configuration, which equalized the overall bundles’ cross-section. Intersections of the wrapping and main fiber strand syntaxes had no adverse effects on the tape draping. Automating the tape impregnation process would enhance the deposition quality and speed in the future.

### Structural performance evaluation

**Figure 9:**
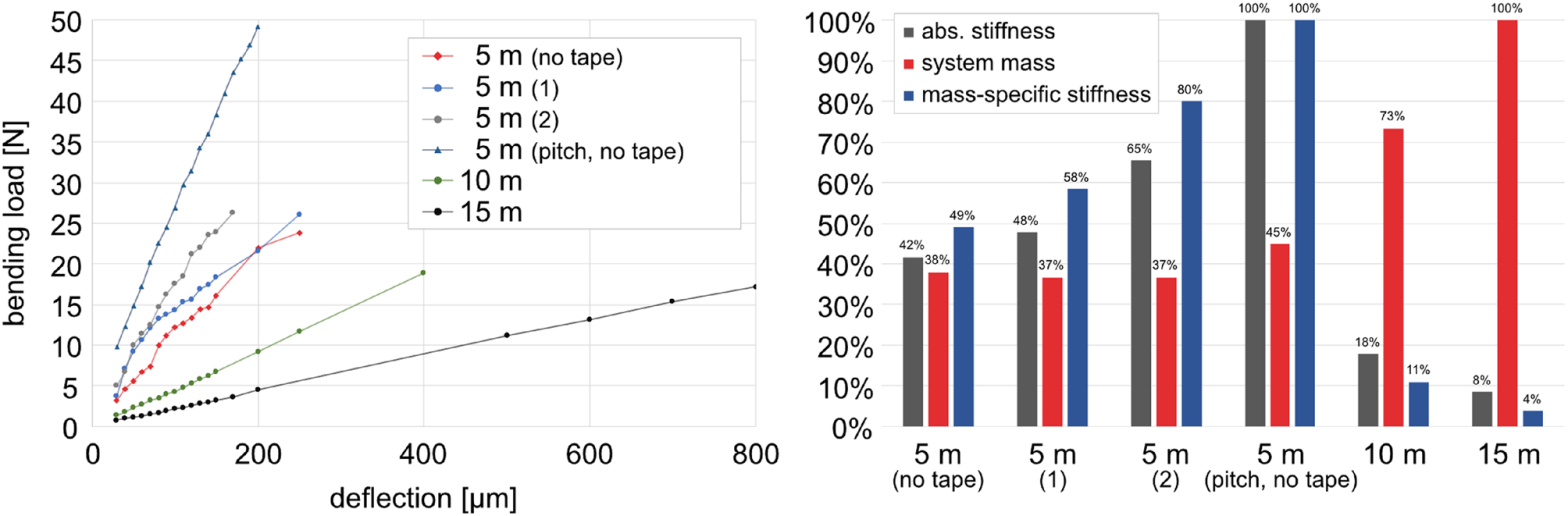
Results of the mechanical testing of the case study samples. Left: Load-deflection measurements. Right: Structural performance analysis of the case study samples, each normalized to maximum.

In accordance with the testing procedure outlined in the “Testing procedure” section, load-deflection measurements, see Fig. 9 left, were conducted and subsequently compared with the system masses of the trusses, see Fig. 9 right.

#### Fabrication deviations

A comparison between samples 2 and 3, see Table 3, indicates fabrication deviations associated with differing fiber tension. Sample 2 was fabricated with lower fiber tension for the deposition of rovings, while the fiber tension for the tape remained consistent across both samples. Notably, this deviation has minimal impact on the necking, which is primarily influenced by the fiber tension ratio between the main strands and the wrapping. Furthermore, both samples exhibit similar system mass, fiber mass ratio, leakage, and actual length. The average FVR of 48.30% ± 2.02% was measured by different state-of-art methods^41,43^. Analyzing the raw data, see Fig. 9 left, stiffness remains similar up to approximately 12.5 N before discrepancies occur. This coincides with the dead weight of the components. As anticipated, the overall structural stiffness is reduced for the sample with lower fiber tension, presumably indicating the beginning of imminent global buckling. As the impact of fiber tension deviation outweighs the impact of the pitch tape reinforcement, see “Impact of the tape reinforcement” section, the importance of a fiber tension monitoring and control system for automated applications is underscored^20^.

#### Impact of the tape reinforcement

The lightweight potential of the pitch tape reinforcement becomes evident from samples 1 and 3, as they were fabricated at similar fiber tension levels. Based on the testing, the implementation of the tape significantly improves the mass-specific bending stiffness of the structures by 57.1%, but adds only 1.4% to the system mass. This is attributed to the tape being strategically positioned at the outer regions of the main fiber strands’ cross-sections, thereby significantly impacting the bending resistance moment. However, the gain in tensile stiffness may be smaller, calculated to be only 16.6% based on the rule of mixtures and the nominal cross-sectional ratio between rovings and tape. Furthermore, the circularization of the cross-section of the main fiber strands, which occurs automatically during the deposition of the tape, contributes to the system’s bending stiffness and also improves the buckling resistance of the structure’s members.

#### Impact of the pitch rovings

The impact of substituting the PAN-based rovings with the pitch-based rovings can be observed from samples 1 and 4. As the pitch carbon fibers have a 38.8% higher density than the PAN-based fibers, also the trusses’ total system mass was increased, as the same amount of rovings (not linear density) was applied. But due to the contribution of the other contained elements, the truss’s mass gain was quantified to be only 17.9%. Despite the increase in system mass, the increment in absolute stiffness by 140% and in mass-specific stiffness by 104% is significant. It is important to note that further adjustments in the fabrication process may further enhance the stiffness, see “Fabrication implications” section. Neglecting potential differences in tensile and bending modulus of the material, the sample currently only reaches 65% of the expected nominal increase based on the tensile data sheet values. Once the fabrication system is properly adjusted for pitch fibers, an even higher impact can be anticipated.

#### Impact of the scaling

The scaling effect on stiffness can be evaluated based on the analysis of samples 3, 5, and 6 by using the actual lengths of the samples rather than the nominal lengths. Concerning the other impact factors discussed above, this assessment is particularly relevant for evaluating the feasibility of the sunshade concept. Scaling can only be accurately investigated at full scale, as material and geometry interaction cannot be accurately scaled. Based on the case study, the increase in length leads to a disproportional loss in stiffness which can be approximated using the equation $k = 530,176,882\,L^{ -1.744}$ where *k* represents the absolute stiffness [N/mm] and *L* denotes the sample length [mm]. The trusses have a linear density of approximately 222.1 ± 17.9 g/m in terms of composite mass, which slightly decreases with increasing length.

### Finite element analysis and prediction

This study contains several FE analyses: Initially, the experimental testing was replicated to reveal fabrication influences, subsequently, the proposed planetary sunshade satellite’s structural concept was analyzed.

#### Experimental testing replication

The aim of the FE analysis in this section was to replicate the experimental testing of the case study in order to identify fabrication influences by comparing expectation and experimental results. The modeling approach was to describe the samples as volume bodies including as many measured parameters of their real counterpart. Therefore, the simulation results of samples 2 and 3 are different. The parameters cover fiber volume ratio, length of the internodal regions, and cross-sectional perimeters along the truss, see Fig. 8. It was also incorporated which crossings of the lateral cross-bracing were structurally connected. Further geometrical parameters were idealized, e.g., the layering and hooking of fiber bundles as well as their cross-sectional shape. The material parameters were calculated based on their data sheets values by using the rule of mixture. Loading and fixation was according to the experimental test setup, see Fig. 6.

**Figure 10:**
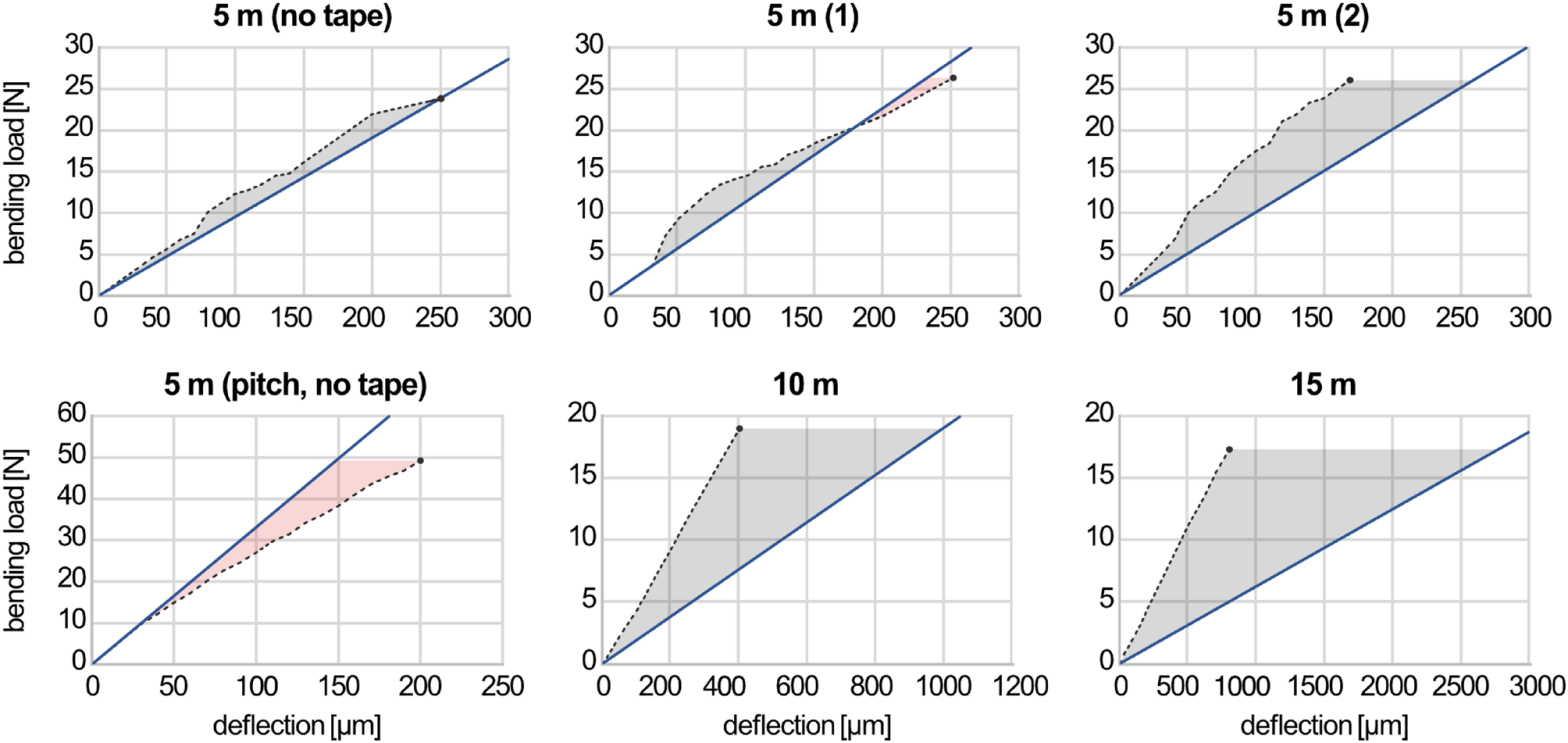
Experimental testing replication. The solid line represents the simulation results, the dashed line represents the testing results, the gray areas show regions where the simulation underestimated the stiffness of the structure, whereas red areas indicate region with overestimated stiffness. Results highlighting the relation between the experimental and predicted structural/material behavior due to fabrication influences. For the calibration factors of the global simulation models however refer to Fig. 11. The ratios do not match with the ones in Fig. 11 due to the different degrees of model simplification.

In general, the simulation results underestimate the structural stiffness of the trusses, especially for increasing truss length, see Fig. 10, highlighting the necessity of simulation calibrations by means of experimental measurements. Only the structural stiffness of the pitch-based carbon fiber truss was significantly overestimated, confirming that here fabrication methods need further improvement to harness the full potential of the pitch-based fibers in CFW. The same is true for higher deflections of sample 2, emphasizing the reduced fiber tension during fabrication.

#### Satellite’s structural concept evaluation

The FE analyses in this section focus on the planetary sunshade satellite’s structural concept, in the following also referred to as global design, see Fig. 2, in order to give a first estimate of the deformations of the structural system. For this purpose, a multi-step process is carried out: First, the experimental testing of the case study is again replicated but by deploying the sample modeling approach used later for the global design. Second, calibration factors are extracted and extrapolated to the required beam lengths for the global design. Finally, the global design is modeled a simple beam framework to obtain the maximum node displacements for different fiber composite materials and satellite’s edge lengths.

In order for the calibration factors to be descriptive, the modeling for the case study replication and the satellite’s structural concept must be identical. Therefore, the entire truss of each sample was simplified as a single beam element, which boundary conditions matching the experimental setup. However, the cross-section for both cases was simplified as an annulus, with an arbitrarily defined outer-diameter-to-wall-thickness-ratio of 100/1, where the outer diameter matches the nominal edge length of the truss. The boundary conditions of the global design model included an even distribution of the load along all nodes at the satellite’s edge and a complete fixation of the two center nodes at the satellite’s bus.

**Figure 11:**
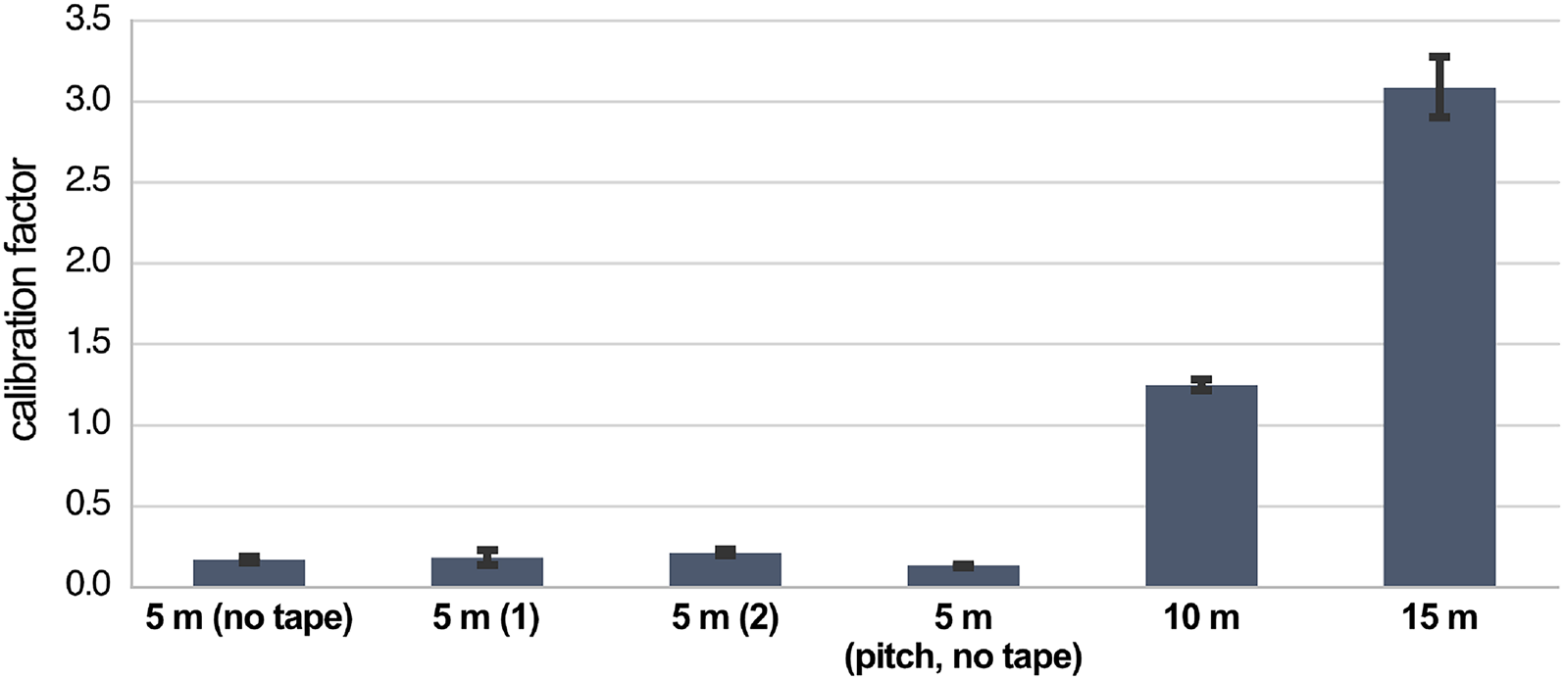
Elasticity calibration factors for all case study samples and load cases. Values for samples 1, 2, and 4 shown for completeness only. The ratios do not match with the ones in Fig. 10 due to the different degrees of model simplification. As the model includes also geometrical simplifications these results do not allow any conclusions to be drawn about the material behavior, for this refer to Fig. 10.

The calibration is approached by directly adjusting the elasticity of the beam segments, rather than adjusting the resulting deformations. This allows the scaling effect found in the case study to be taken into account. The elastic modulus of the material was calculated by the rule of mixtures based on the given data sheet values of each material and the measured FVR from the case study. A calibration factor was then applied to the elastic modulus of the material, which was iteratively adjusted until the resulting deflection matches the experimentally determined one. As the determination of the calibration factor was performed individually for each load case the procedure was automated using a simple linear interpolation that converged sufficiently fast. Although the calibration factors were determined for all load cases, see Fig. 11, only samples 3, 5, and 6 were considered for quantifying the scaling effect.

**Figure 12:**
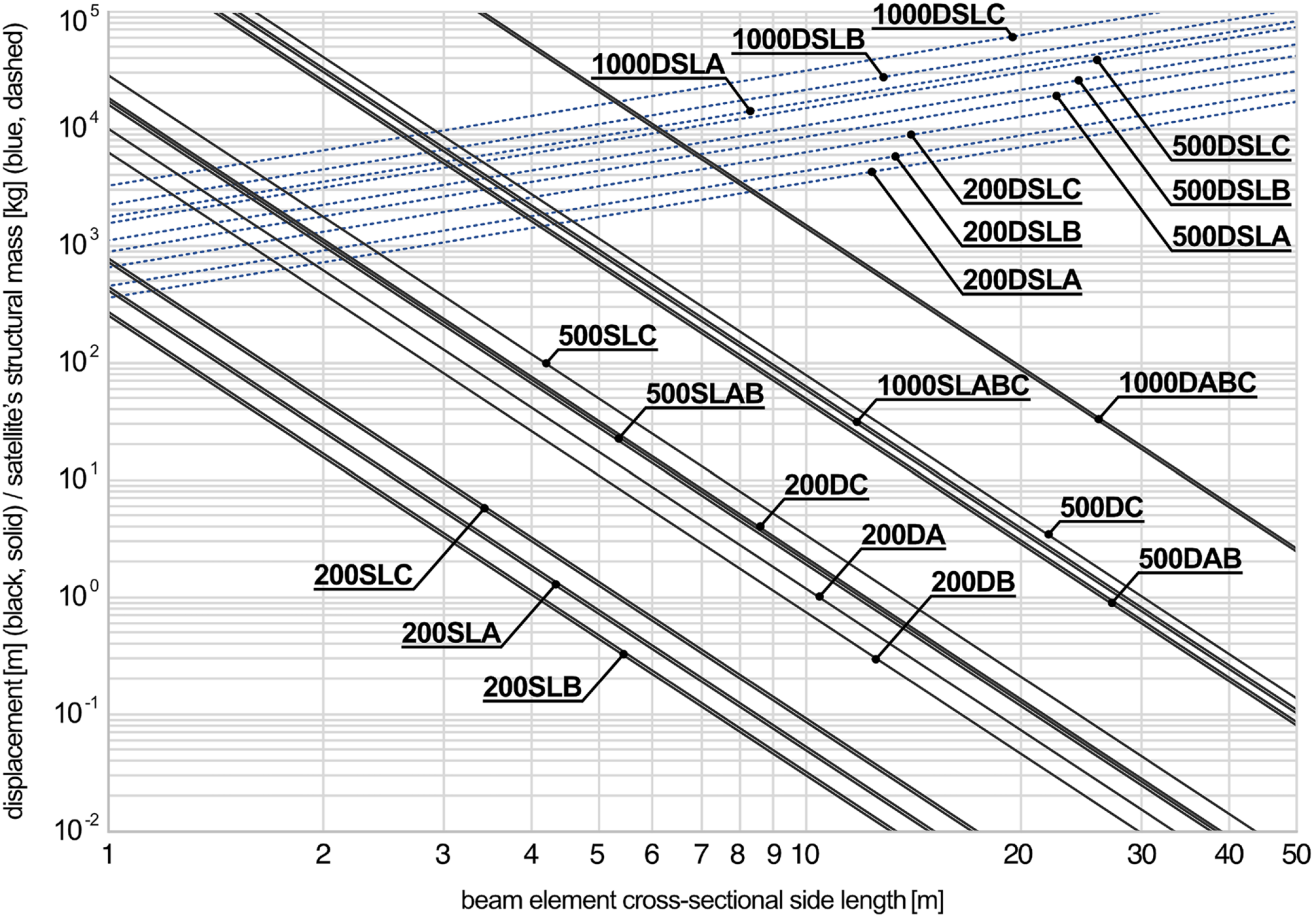
FE analysis results (log–log scale) of the planetary sunshade satellite’s structural concept for several scenarios. Displacement graphs are decreasing, the structural mass graphs are increasing with beam element cross-sectional side length. Labels combine the (**1000**, **500**, **200**) satellite’s edge length in m; the load cases, including **(D)** atmospheric drag in SSO, **(S)** solar radiation pressure in GSO, and **(L)** solar radiation pressure in SEL1 halo; and fiber composite material, including **(A)** PAN-based carbon with pitch-based carbon reinforcement tape and epoxy matrix, **(B)** pitch-based carbon fibers with epoxy matrix, and **(C)** basalt fibers with mineral matrix. Some graphs are overlapping. The beam element cross-sectional side length refers to the side length of the triangular nodals (0.3 m in the case study).

For the extrapolation of the calibration factors representing the scaling effect a quadratic polynomial was used. It was found as $K = 0.0101176780~L^2 + 0.0116911288~L - 0.131827599$ where *K* represents the calibration factor to be applied to the beam’s nominal fiber/matrix-composite elasticity and *L* denotes the beam length [m]. The underestimation of the material stiffness in the simulation increases disproportionately with the beam length. The maximum segment length in the 1 km^2^ planetary sunshade satellite’s structural concept is 280 m, however, the measurements cover the 5 to 15 m range only. Hence, the data basis for the extrapolation is limited in size and relevance due to the practical restrictions of the case study. Therefore, the authors would like to already note at this point that the significance of the following simulations is correspondingly limited. However, in the next step, the segment lengths of the 200 m, 500 m, and 1 km edge length global designs were extracted and the individual calibration factors were automatically assigned to the model. The model has a uniform cross-section and neglects all satellite sub systems except the actual truss support structure. The maximum displacement of all nodes and segment midpoints was then evaluated based on the expected highest loads per orbit and edge lengths, see Table 1, including (A) the PAN-based carbon with pitch-based carbon reinforcement tape and epoxy matrix, (B) pitch-based carbon fibers with epoxy matrix, and (C) basalt fibers with mineral matrix. The last material combination provides an outlook on in-situ resource utilization (ISRU) concepts. A FVR of 50% was assumed for the basalt fibers, for the other material combination the characteristic FVRs from the case study were used. Parameters for the basalt fiber material system were taken from literature^60^. The resulting displacements and corresponding satellite’s structural mass can be displaced best in log–log scale, see Fig. 12, resulting in several parallel linear graphs.

For the beam element cross-sectional side length a range of 1 to 50 m was set, as values outside this range seem irrelevant for space applications in the 200 to 1000 m edge length scale. The resulting displacements and masses are therefore distributed over several orders of magnitude and reach unrealistic values predominantly in the peripheral regions of the plot, which are nonetheless presented for completeness. Flights in low Earth orbits, see SSO in Fig. 12, are only relevant in an earlier phase of the sunshade development timeline, where smaller edge lengths than 200 m are to be expected. The presented case study involving CFW is also more relevant to concepts using ISMA for larger satellites made from in-situ resources. To name just a few exemplary value pairs from Fig. 12: For the 1000 m edge length sunshade a maximum node displacement around 65 m can be expected for both solar radiation pressure load cases and at a beam element’s side length of 10 m. At 30 m side length the displacements drops repeatedly to values around 80 cm. The total structural mass of 28 tons for the 10 m and the 83 tons for the 30 m variation, are relativized when expressed as foil-area-specific values of 28 and 83 g/m^2^ respectively. Looking at the mass-specific inverted displacements, a general trend towards more efficient structures can be found for increasing beam element side length, which can be explained by the gain in stiffness due to an increased profile’s bending moment.

Besides a first estimation of the displacement for different scenarios, the FE analyses reveal the relative strength of the various influencing factors by looking at the clustering of the graphs. The edge length has the most important impact for both displacement and system mass. The second strongest influence is the load level. For smaller designs the influence of the material selection makes a difference for the displacement, but can be neglected for larger designs. Regarding the system mass the load case is negligible. Primary the satellite’s edge length and secondly the material selection are relevant influencing factors. As expected, for all scenarios the PAN-based carbon with pitch-based carbon reinforcement tape and epoxy matrix is the lighter than the pitch-based carbon fibers with epoxy matrix. The basalt fibers with mineral matrix design is the heaviest, whereby the mass increase from pitch-based carbon to basalt fibers is about twice as large as from the PAN-based to the pitch-based carbon fiber material system. Interestingly the 500 m basalt fiber material system design is nearly as heavy as the 1000 m PAN-based carbon fiber material system design. This underlines the effectiveness of a strategically positioning of the pitch-based carbon reinforcement tape within the fiber bundles cross-section. However, when optimizing for minimal displacement the pitch-based carbon fiber material system is the best option. Using a basalt fiber material system for ISRU purposes significantly increases the mass of the system, which would correspondingly increase the logistics required.

The manufacturing influences identified in the case study are inherited by the FE analyses in this section only in the form of FVR, the fiber tension and necking effects are not considered. The calculated maximum displacement of the truss is not equal to the deflection of the foil, because in the current concept it is only fixed at the perimeter nodes of the satellite. In order to make a statement about the deformation of the foil, the mechanical properties of the foil have to be taken into account, which is beyond the scope of this study. Theoretically, no deformations of the foil are to be expected if all edge points of the truss are uniformly displaced, which could be a design criterion for the further optimizing of the satellite’s structural concept. For the foil, two influences from the structural system are relevant: differential displacements perpendicular to the foil and displacements within the foil plane. The latter are significantly smaller than the total displacements plotted in Fig. 12.

Although the authors do not define a threshold for the maximum tolerated node displacement, the current estimation in Fig. 12, reveals that expected deformations for reasonable beam element cross-sectional values lie in a range, which allows the concept to be evaluated as being plausible for future implementation; especially with regard to the expected improvements with improved adaptation of the fabrication system to pitch carbon fibers as well as with a further topology optimization of the global design. Especially relevant for the further development of the planetary sunshade concept itself is that with increasing edge length of the satellites the material selection becomes less decisive, which emphasizes that ISRU in combination with on-orbit manufacturing represent a plausible approach to future research.

## Conclusions

In this study, a comprehensive investigation of a structural system fabricated by coreless fiber winding and designed for long-span fiber composite lightweight satellite structures was conducted. The foil support structure of a planetary sunshade satellite was used as a predestined case study. Initially, the study proposes a novel global structural system for the satellite as well as a modular component-level truss system.

Within a laboratory-scale case study, six truss samples were manufactured and subjected to custom bending deflection testing. The experimental investigations allowed for the identification of the key factors governing the structural performance of the system, including its scalability, fabrication deviations, and fiber composite material selection. The investigation on the effect of scalability in truss length was investigated between 5 and 15 m form-factors revealing the disproportional loss in stiffness with increasing length. The fabrication deviations were identified, as a loss in stiffness resulting from decreased fiber tension during winding. The impact of fiber tension on necking of the structures was found to be minimal. However, the importance of fiber tension monitoring and control must be emphasized. The incorporation of pitch-based carbon reinforcement tape onto the PAN-based carbon fiber strands showed a remarkable improvement in the trusses’ mass-specific bending stiffness, with a negligible increase in system mass. Exclusively deploying pitch-based fibers, despite a slight increase in system mass, presented a substantial increment in absolute as well as mass-specific stiffness. However, it is expected that the stiffness can be further improved by process adaptations that allow higher fiber tension for pitch fibers. The experimental results suggest that the modular truss system can be adapted and scaled to fit diverse structural requirements, affirming its potential for real-world applications.

Subsequently, two numerical analyses were conducted. The first involved a high-detail modeling of the truss samples revealing manufacturing influences by comparing anticipated and actual mechanical performance. The structural stiffness was underestimated by the numerical replication of the experimental testing, especially for longer trusses. For the pitch-based carbon fiber truss stiffness was significantly overestimated, which confirms the need for CFW fabrication system improvements targeted towards pitch. The second numerical analysis comprised a simplified modeling approach, calibrated by the results from the experimental case study, providing an estimate of mechanical performance and system mass for the planetary sunshade structural design concept. Here, future research might need to define maximum tolerable limits for the deformations of the satellite’s structure and foil system as well as include joint elements in the FEM model. Furthermore, analyses of local and global buckling^61^ must be performed while considering fabrication deviations. Nevertheless, the range of expected deformations, based on reasonable beam element cross-sectional values, were found to be encouraging for the feasibility of the planetary sunshade concept. Notably, the reduced significance of material selection at increasing foil edge lengths emphasizes the viability of ISRU approaches using basalt and mineral matrix material systems, especially when combined with ISMA as a promising avenue for future research. Consequently, this study has demonstrated the potential of coreless filament winding for the manufacture of large-scale satellite structures.

## Data Availability

The datasets used and/or analyzed during the current study are available from the corresponding author upon request.
